# Novel Truncating and Missense Variants in *SEMA6B* in Patients With Early-Onset Epilepsy

**DOI:** 10.3389/fcell.2021.633819

**Published:** 2021-05-04

**Authors:** Song Xiaozhen, Yuan Fan, Yuan Fang, Lan Xiaoping, Jia Jia, Xu Wuhen, Tang Xiaojun, Shen Jun, Chen Yucai, Zhang Hong, He Guang, Wu Shengnan

**Affiliations:** ^1^Laboratory of Molecular Diagnosis, Shanghai Children’s Hospital, Shanghai Jiao Tong University, Shanghai, China; ^2^Bio-X Institute, Key Laboratory for the Genetics of Developmental and Neuropsychiatric Disorders, Ministry of Education, Shanghai Jiao Tong University, Shanghai, China; ^3^Shanghai Key Laboratory of Psychotic Disorders, Shanghai Institute of Mental Health, Shanghai Jiao Tong University, Shanghai, China; ^4^Department of Neurology, Shanghai Children’s Hospital, Shanghai Jiao Tong University, Shanghai, China; ^5^Fuxiang Gene Engineering Research Institute, Shanghai, China; ^6^Department of Pathology, Brigham and Women’s Hospital, Harvard Medical School, Boston, MA, United States

**Keywords:** *SEMA6B*, epilepsy, nonsense, gene, missense variant

## Abstract

Progressive myoclonic epilepsy (PME) is a rare neurodegenerative disease, characterized by myoclonic seizures and tonic clonic seizures, with genetical and phenotypical heterogeneity. The semaphorin 6B (*SEMA6B*) gene has been recently reported a causal gene of PME. Independent studies are warranted to further support these findings. Here we report that one nonsense variant in NM_032108.3 exon17 c.2056C > T (p.Gln686^∗^) and one missense variant in exon14 c.1483G > T (p.Gly495Trp) of *SEMA6B*, both occurring *de novo*, underlie early-onset epilepsy with variable severity and different response to treatment in two patients. *In vitro* analyses have demonstrated that the nonsense variant, p.Gln686^∗^, results in a truncated protein with remarkably increased expression compared to that of the wild type. The truncated protein presented more homogeneous and failed to locate in the plasma membrane. The missense variant p.Gly495Trp affects evolutionarily conserved amino acid and is located in the sema domain, a key functional domain of SEMA6B. It was predicted to perturb the SEMA6B function by altering the tertiary structure of mutant protein, although neither change of protein length and expression nor difference of cellular distribution was observed. Co-immunoprecipitation studies have demonstrated that both variants influence protein binding of SEMA6B and PlxnA2 with varying degrees. Our results provide further evidence to support the initial findings of *SEMA6B* being causal to epilepsy and indicate that mediating Semaphorin/Plexin signaling is the potential mechanism of the SEMA6B-related disease.

## Background

Progressive myoclonic epilepsies are a group of more than 10 rare types of epileptic syndrome. For all types of progressive myoclonic epilepsy (PME), it usually starts in childhood or adolescence, and a few can be infancy onset (Genton et al., 2016; Turnbull et al., 2016). The main clinical manifestations of PME are frequent myoclonic seizures, often accompanied by generalized tonic clonic seizures and other forms of seizures, and progressive neurological dysfunction including cerebellar ataxia, dementia, etc. While electroencephalogram (EEG) images showed spike-and-wave discharges that are typical in the epilepsy phenotype, treatment failure, and progressive neurological symptoms all point to the diagnosis of PME.

Latest advances in whole exome sequencing and analytics have led to tremendous progress in the discovery of novel genes underlying the etiologies of epilepsy, which is clinically and genetically heterogeneous. The yield of epilepsy genetics has enabled molecular diagnoses of previously unidentifiable epilepsy and contributed to the development of new targeted treatments (Ellis et al., 2020; Perucca et al., 2020). A recent study has revealed a new genetic cause of PME (Hamanaka et al., 2020). Hamanaka et al. have reported four *de novo* truncating variants, c.1950_1969dup (p.Arg657Profs^∗^35), c.1976_1982 (p.Ala659Valfs^∗^24), c.1982_1991 (p.Gly661Alafs^∗^21), and c.1991 (p.Gly664Alafs^∗^21), within the *SEMA6B* (OMIM# 608873) gene that encodes the semaphorin 6B in five unrelated patients, associating *SEMA6B* with PME-11 (EMP11, OMIM#618876). The reported patients of EMP11 experienced additional neurological symptoms and signs during the first and second decades, including pyramidal, extrapyramidal, and cerebellar symptoms such as spasm, loss of independent walking ability, myoclonus, tremor, and ataxia. Cognitive impairment is serious; patients can only say a few words or non-verbal mumbling. Interestingly, all truncating variants detected in this gene are located within NM_032108.3 exon17, the last exon of *SEMA6B*. No truncated variations in exon17 of the *SEMA6B* gene were observed in the gnomAD database^1^. These variants were expected to lead to the production of truncated proteins that lack intracellular domains. Hamanaka et al. found that zebrafish with distal truncating variants affecting the intracellular domain of SEMA6B orthologs that escaped from nonsense-mediated mRNA decay [NMD(−)] had defective brain development and were more susceptible to seizures compared to the wild type. In addition, zebrafish with loss-of-function mutations had less severe abnormalities compared to those with distal truncating mutations, indicating that truncating mutations exert a more detrimental toxic effect than loss-of-function mutations. Gain-of-function or dominant-negative effects are speculated to be the disease-causing mechanism of such variants.

Semaphorins are secretory proteins of the plasma membrane characterized by cysteine-rich semaphorin protein domains whose expression and functions are implicated mainly in the development of the nervous system and axon guidance (Yazdani and Terman, 2006). A growing body of evidence has indicated that semaphorins play important roles in cancer (Capparuccia and Tamagnone, 2009; Neufeld et al., 2016). Semaphorin 6B is 1 of the 20 protein family members in humans. The pioneering study by Hamanaka et al. (2020) strongly indicated that *SEMA6B* is a promising disease-causing gene of epilepsy. Semaphorin/Plexin signaling is one of the mechanisms regulating dendrite differentiation, which is included in the pathological features of epileptogenesis (Kokaia, 2011; Cheadle and Biederer, 2014). Plexin-A2 (PlxnA2) has been demonstrated to be a potential interaction partner for SEMA6B in commissural axon guidance (Tawarayama et al., 2010; Andermatt et al., 2014). However, independent confirmations are currently missing, and no *SEMA6B*-linked epilepsy cases have been reported in other populations. Missense variants of SEMA6B have not been observed in patients with epilepsy, which are known to often cause disease through gain-of-function or dominant-negative effects. Here we present complementary data showing that novel variants occurring *de novo* in SEMA6B were found in two unrelated individuals with epilepsy.

## Materials and Methods

### Patients and Genetic Analysis

The two patients in this study were recruited from the Neurology Department of Shanghai Children’s Hospital, and the parents of both patients were included for genetics analysis. Written informed consents for the use of clinical and genetic information were obtained from the parents. This study was approved by the Ethics Committee of Shanghai Children’s Hospital (approval No. 2020R007-F01).

Due to the known efficiency of trio-based whole exome sequencing (TWES) on neurodevelopmental diseases, we performed TWES on the two probands and their parents to elucidate the cause of disease. Peripheral blood samples were collected for genomic DNA preparation. The IDTxGen^®^ Exome Research Panel (IDT, United States) was used for exome capture, and pair-end sequencing was performed on HiseqX10 (Illumia, United States) to obtain 2 × 75 reads. Fastq data of all subjects were analyzed to identify single nucleotide variants, small insertions, and deletions, and copy number variants using an in-house pipeline. The American College of Medical Genetics and Genomics/Association for Molecular Pathology (ACMG/AMP) recommendations were used to interpret the clinical significance of susceptible variants (Richards et al., 2015).

### Protein Structure Modeling

The I-TASSER (Iterative Threading ASSEmbly Refinement) online server has been used to predict the effect of the missense variant p.Gly495Trp on the tertiary structures of SEMA6B (Yang et al., 2015). The best predicted structure with the maximum confidence score (C-Score) was selected for further analysis.

### Plasmid

The N-terminal EGFP tagged cDNA clone of the *SEMA6B* transcript NM_032108.3 (pEGFP-C1-human SEMA6B) expression plasmid was synthesized and constructed by Shanghai Jushun Biotechnology Company. The schematic representation of the wild-type (WT) SEMA6B construct is shown in Supplementary Figure 1. SEMA6B mutations c.1483G > T (p.Gly495Trp) and c.2056C > T (p.Gln686^∗^) were introduced into the WT isoform using a site-directed mutagenesis kit (QuikChange Lightning Site-Directed Mutagenesis Kit from Agilent Technologies, Santa Clara, CA, United States). The whole sequence (WT or mutants) was confirmed by Sanger sequencing. The pCMV-EGFP- C1 vector was used as a marker of transfected cells. The primers used in site mutation are shown in Supplementary Table 1. Myc-DDK-tagged human plexin A2 (RC221024) expression vectors were purchased from OriGene Technologies (Rockville, MD, United States).

### Cell Culture and Plasmid Transfection

To characterize the function consequence of the *SEMA6B* variants detected in the present subjects, wild type (SEMA6B^WT^) and two mutant plasmids (SEMA6B^Gly495Trp^ and SEMA6B^Gln686*^) were prepared for cell transfection. Human embryonic kidney cells (HEK)- 293T cells were cultured in Dulbecco’s Modified Eagle’s Medium (DMEM) (Gibco, United States) supplemented with 10% fetal bovine serum (FBS) (Gibco, United States) and with 1% antibiotics (Gibco, United States) at 37°C in a humidified 5% CO_2_ atmosphere incubator. Transient transfection was performed by a FuGENE^®^ HD reagent (Promega, United States) according to the manufacturer’s recommendations. Briefly, the cells were plated the day before transfection at a density of 5 × 10^5^ cells per well of a 6-well plate, and 24 h later, plasmid DNA was added at 3.3 μg/well diluted with 155 μl Opti-MEM (Gibco, United States) and mixed with 9.9 μl of FuGENE.

### Immunoblot Analysis of Wild Type and Mutant SEMA6B

HEK-293T cells were collected and lysed with a RIPA lysis buffer (Beyotime Biotechnology, China) 48 h after transient transfection. Total cell lysates were mixed with a 5 × SDS loading buffer (Beyotime Biotechnology, China), boiled for 10 min. Subsequently, sodium dodecyl sulfate-polyacrylamide gel electrophoresis (SDS-PAGE) analyses were performed according to the standard protocols using 8% polyacrylamide gels. Proteins were transferred onto PVDF membranes (GE Healthcare, Germany). After blocking with 5% skim milk, PVDF membranes were incubated with primary antibodies overnight at 4°C, followed by incubation with HRP-conjugated antirabbit or antigoat (Jackson, United States) secondary antibodies at room temperature for 1 h. Blots were developed using the ECL reagent (Share-bio, China). All experiments were done in triplicates after optimal working conditions were determined. Primary antibodies used were rabbit anti-Actin (Cell signaling technology; 1:5000) and goat anti-SEMA6B (R&D Systems, Germany; 1:1000).

### Subcellular Distribution of SEMA6B

To analyze the subcellular distribution of SEMA6B^WT^, SEMA6B^Gly495Trp^, and SEMA6B^Gln686*^, we used HEK-293T cell lines transiently transfected with the N-terminally EGFP-tagged SEMA6B (WT or mutant) after fixation in 4% paraformaldehyde; the cultured cells were incubated with DAPI for 5 min at room temperature. Confocal images were acquired with a Super-resolution Multiphoton Confocal Microscope using a 100× objective (Leica, Germany).

### Coimmunoprecipitation

HEK-293T cells (1 × 10^6^ cell/ml) were transfected with Myc-tagged PlxnA2 and EGFP-tagged SEMA6B. After 48 h, cells were lysed with an NP-40 buffer containing protease inhibitor cocktail; the main components are 50 mM Tris (pH 7.4), 150 mM NaCl, 1% NP-40, and multiple inhibitors (Beyotime Biotechnology, P0013F, Beijing, China). Then the cell lysates were immunoprecipitated (IP) with anti-GFP antibody overnight at 4°C (Abcam, MA, United States), where each IP was carried out using 5-μg antibody and 500-μg protein. After that, each IP was treated with A/G agarose beads for 4 h at 4°C (Santa Cruz Biotechnology, United States). Beads bound to IP were then washed with a RIPA buffer five times. In the following Western blot assay, the whole-cell lysate was classified into an input group as the positive control, and the Myc antibody was used as the primary antibody to verify Myc-tagged PlxnA2 protein (Cell Signaling Technology, MA, United States).

## Results

### Clinical Presentations of Affected Individuals

**FIGURE 1:**
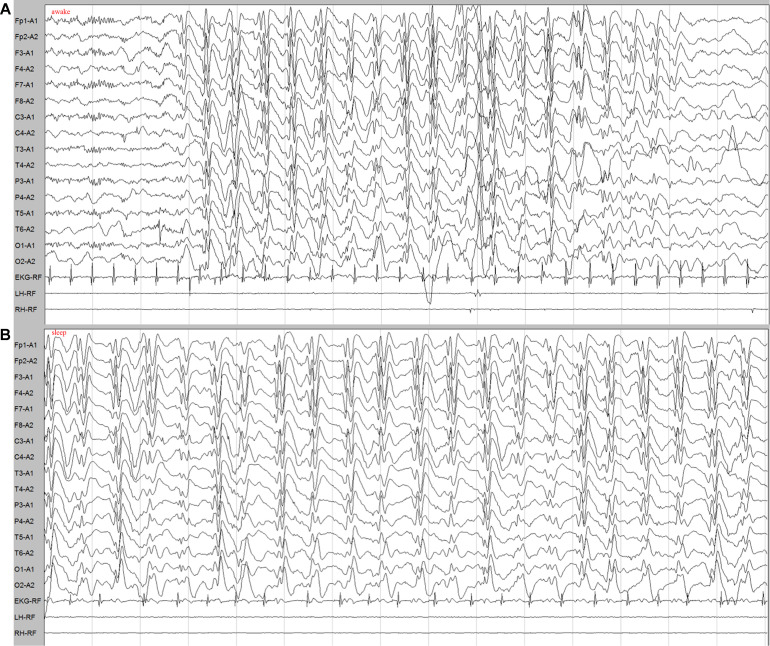
EEG demonstrated generalized slow spike-and-wave complexes (1.6–2 Hz) in the patient. **(A)** Intermittent slow spike–wave discharges during awake. **(B)** Continuous spike–waves during sleep.

Patient one is a currently 4-year-old girl of Chinese origin visiting the neurology department in our hospital with a chief complaint of uncontrolled seizures. She was the only child of the family, born to non-consanguineous and healthy parents without complications at term. Her birth weight was 2,450 g, and the birth length was 47.0 cm. She had mild delayed developmental milestones, with head control at 6 months, sitting independently at 10 months, standing alone at 13 months, and walking with support and speaking consciously at 18 months. The first type of seizure was atonic seizure onset at 2 years old characterized by sudden atonic falls, and the frequency was about three to five times per month. The second type was complex partial seizure. She had no response to voice commands with masticatory movements, a folded-opened left hand, and an unbent left body, which lasted nearly 20 min during the seizure. The frequency of this type was about two to five times per month. The third type was atypical absence seizures, which presented as a sudden loss of consciousness for about 1–2 min. The frequency of this type was about four to six times weekly. The electroencephalogram (EEG) showed generalized slow spike-and-wave (SSW) discharges (1.6–2 Hz), demonstrating intermittent discharges during awake (Figure 1A) and continuous spike–waves during sleep (Figure 1B). Non-specific finding was seen in the MRI images. She started levetiracetam and carbamazepine treatment after the second episode of seizures, which was not effective. Significant motor regression and intellectual regression were noticed with losing speech and becoming unable to walk independently. A negative family history of neurological disease was reported by the parents.

Patient 2 is a 3-year-old boy born to non-consanguineous Chinese parents with no complications. He was referred to the neurological department of our hospital due to the onset of seizures with eye-rolling, cyanotic lips, consciousness lapses, right upper limb jitter, and a gelastic seizure for more than 15 s at the age of 2 years. No obvious abnormality was detected in his brain MRI. The frequency of the seizure is nearly two to five times weekly. Fortunately, he had good response to combination treatment of levetiracetam and sodium valproate with no recurrence of epilepsy. He has apparently normal developmental milestones. No intellectual and motor developmental regressions have been observed.

### Genetic Findings and *in silico* Predictions

**FIGURE 2:**
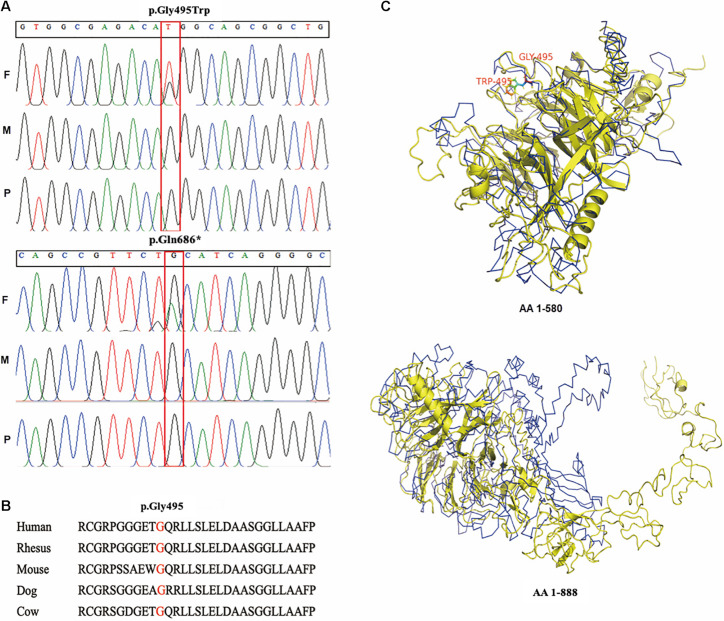
Genetic variants detected in the patients and *in silico* prediction. **(A)** Sanger confirmation of two *SEMA6B* variants in the patients and their parents; **(B)** conservation of p.Gly495 in mammals; **(C)** upper: protein tertiary structure of amino acid residues 1–580; lower: full-length protein tertiary structure (amino acids 1–888). F: father; M: mother; P: proband; AA: amino acid.

After stringent filtering of whole exome sequencing data, rare *de novo* variants in the *SEMA6B* were retained as the only disease-causing variants in the two patients. A heterozygous nonsense variant NM_032108.3: c.2056C > T (p.Gln686^∗^) within exon 17, the last exon of *SEMA6B*, and a heterozygous missense change NM_032108.3:c.1483G > T (p.Gly495Trp) in exon 14 were found in patients 1 and 2, respectively. Both variants were absent from the Genome Aggregation Database (gnomAD) and were confirmed by Sanger sequencing (Figure 2A).

The missense change, p.Gly495Trp, is predicted to be damaged by DANN, FATHMM-MKL, FATHMM-XF, MutPred, MutationTaster, and SIFT4G^2^ and highly conserved in mammals (Figure 2B). It is located in the SEMA domain of the protein, which is an important functional domain of semaphorin. Protein structure analysis has shown that p.Gly495Trp results in the tertiary structure presenting extremely unique between the mutant and the wild type upstream of amino acid residue p.Leu580, and remarkably loose downstream of this location (Figure 2C), indicating an influence on the tertiary structure of amino acids in the last exon.

### Effects on Protein Expression and Cellular Distribution

**FIGURE 3:**
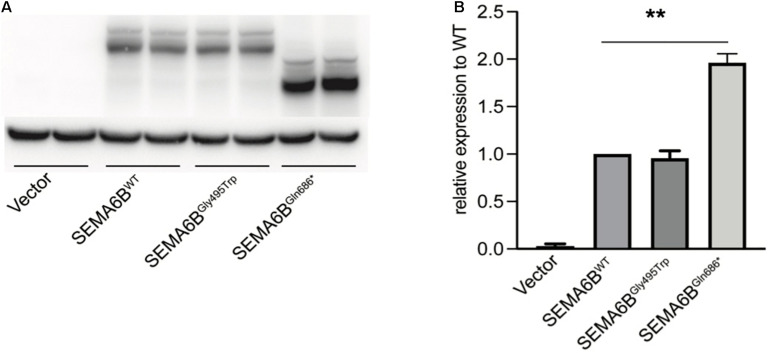
Protein expression of wild type and mutant SEMA6B. **(A)** SEMA6B^Gln686*^ demonstrated a shorter protein and remarkably increased expression compared to wild type. **(B)** Densitometric analysis of SEMA6B. Values were normalized using actin. Three independent experiments were performed for each condition (*N* = 3, ^**^P < 0.05).

Western blot studies of protein extracts derived from transiently transfected HEK-293T cell lines (wild type and two mutations) revealed a different molecular weight pattern. No significant difference of protein length and expression was observed between SEMA6B^WT^ and SEMA6B^Gly495Trp^. However, SEMA6B^Gln686*^ showed a smaller molecular weight pattern compared to wild type, to our surprise, the protein expression of which is significantly higher than that of the wild type (Figure 3A). SEMA6B^Gln686*^ was two-fold higher than SEMA6B^WT^ (*P* < 0.05; Figure 3B).

**FIGURE 4:**
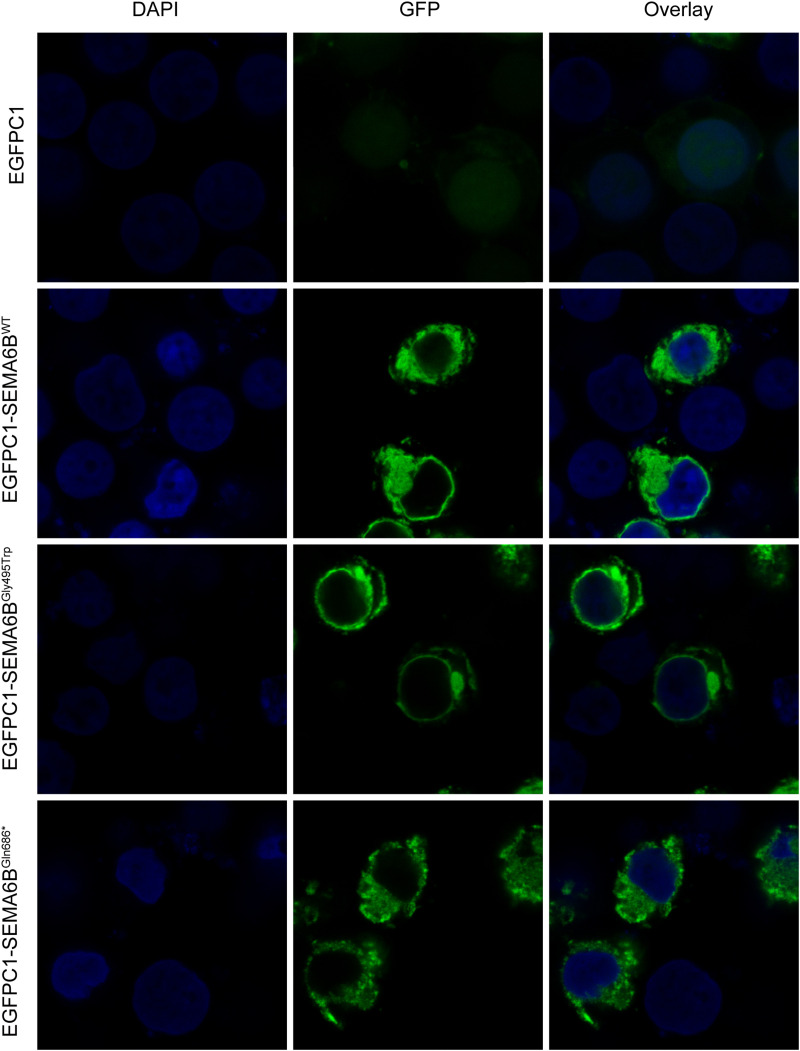
Protein localization of wild type and mutant SEMA6B in plasma membrane and perinuclear membrane. Subcellular distribution of SEMA6B^Gln686*^ failed to locate in plasma membrane.

Furthermore, we studied HEK-293T cells expressing the N-terminal WT or mutant EGFP-SEMA6B fusion protein using high-resolution confocal microscopy. WT EGFP-SEMA6B showed plasma membrane expression and accumulations in perinuclear membrane systems. Meanwhile, SEMA6B^Gly495Trp^ demonstrated apparently similar subcellular localization as the wild type. In contrast, the subcellular distribution of the SEMA6B^Gln686*^ appeared more homogeneous and cannot locate in the plasma membrane (Figure 4).

### Effects on Protein Interaction of SEMA6B and PlxnA2

**FIGURE 5:**
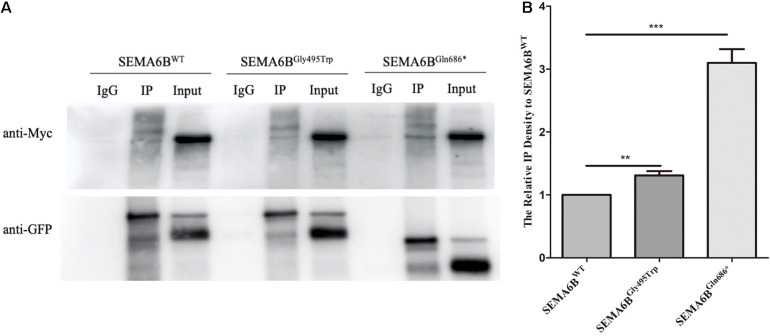
Protein binding of PlxnA2 and SEMA6B. **(A)** The pulled-down Myc-PlxnA2 increased in SEMA6B^Gly495Trp^ and SEMA6B^Gln686*^compared to wild type as shown by Co-IP analysis. **(B)** Densitometric analysis of Myc-PlxnA2. Values were normalized using GFP. Three independent experiments were performed for each condition (*N* = 3, ^**^P < 0.05, ^***^P < 0.01).

To test whether these two *SEMA6B* mutations would change the physical interaction with PlxnA2, HEK-293T cells were transfected with expression vectors for Myc-tagged PlxnA2 and EGFP-tagged SEMA6B. Lysates were subjected to anti-GFP immunoprecipitations, and eluates were separated by SDS-PAGE and immunoblotted with anti-Myc antibodies. As indicated in Figure 5A, the interaction between SEMA6B^WT^ and PLxnA2 was observed, which was similar to previous observations. SEMA6B^Gly495Trp^ has presented a stronger protein binding with PlxnA2 than SEMA6B^WT^. Meanwhile, the truncated protein SEMA6B^Gln686*^ increased binding with PlxnA2 more significantly than SEMA6B^Gly495Trp^ did. The pulled-down Myc-PlxnA2 increased by 1.3-fold and three-fold in SEMA6B^Gly495Trp^ and SEMA6B^Gln686*^, respectively, compared with SEMA6B^WT^ as shown by densitometric analysis (*P* < 0.05; Figure 5B).

## Discussion

To the best of our knowledge, p.Gln686^∗^ and p.Gly495Trp within *SEMA6B* are the first reported variants found in patients with epilepsy, as well as being the first *SEMA6B* variants found in Chinese epilepsy patients. Our results have supported the findings of the initial genetic study on *SEMA6B* and epilepsy (Hamanaka et al., 2020).

Recently, *de novo* truncating variants of *SEMA6B* uniquely identified in the last exon were reported in five unrelated individuals affected with myoclonic epilepsy, in which the protein truncation in the last exon was found to cause defective development of brain neurons and enhanced seizure behavior in the zebrafish model (Hamanaka et al., 2020). Here, we report two novel variants including one nonsense variant in exon17, which is the last exon of SEMA6B, and, interestingly, one missense change occurring within the 14th exon in two unrelated patients with early-onset epilepsy. Variable severity and different response to epilepsy drug therapy have also been observed in these patients. Of note, *in vitro* studies showed that p.Gln686^∗^ resulted in a truncated SEMA6B protein with significant increased expression, which could be toxic and caused abnormal subcellular distribution in the cell model. NMD-escape variants causing increased expression of truncated proteins are a well-known mechanism for human diseases (Poli et al., 2018; Litchfield et al., 2020). The deleterious truncated protein caused by the nonsense variant has resulted in increased binding of PlxnA2 and eventually more severe clinical presentations. On the other hand, the missense change p.Gly495Trp did not demonstrate significant difference of either protein length or expression level as expected. The milder manifestation of epilepsy and positive response to drug treatment might attribute to the tender effect caused by the missense variant. Interestingly, *in silico* analyses showed that this missense variant exerted an influence on the protein fold downstream of amino acid residual 580, which corresponds to the last exon of SEMA6B. The potential structural change may result in abnormal function in the signaling pathway. Molecular and genetic studies suggested that signaling pathways are necessary for GABA interneuron development, which is an activity that is dependent on the member of the semaphorin family of signaling proteins that are disrupted in epilepsy (Gant et al., 2009). Semaphorins are known to interact with partners and regulate the signaling pathway. Sema3A and sema3F regulating the migration of GABAergic neurons in the developing neocortex have been reported (Tamamaki et al., 2003; Li et al., 2019). In addition, Daniel et al. discovered a role for Sema4D as a positive regulator of functional GABAergic synapse formation, and Sema4D could suppress epilepsy (Acker et al., 2018). As one of the members of the semaphorin family, SEMA6B is likely to be involved in the onset of epilepsy. Based on the results and known signaling pathways, it is speculated that the variants in SEMA6B found in our study may influence the Semaphorin/Plexin signaling. Our further studies on the interaction of SEMA6B and PlxnA2 have supported the fact that nonsense variants lead to more increased protein interaction of SEMA6B and PlxnA2, which corresponded to more severe clinical phenotypes in patient 1. The results indicated that both variants caused disease via Semaphorin/Plexin signaling with varying degrees.

In conclusion, the present findings confirm that *de novo* heterozygous variants of *SEMA6B* underlie an autosomal dominant type of myoclonic epilepsy with variable severity and different response to anti-epileptic treatment. Our *in vitro* studies indicated that SEMA6B causes epilepsy with mediating Semaphorin/Plexin signaling. More patients with SEMA6B variants and further *in vivo* studies are required to delineate the pathophysiological mechanism of the newly recognized disorder.

## Data Availability Statement

The raw data supporting the conclusions of this article will be made available by the authors, without undue reservation.

## Ethics Statement

The studies involving human participants were reviewed and approved by the Ethics Committee of Shanghai Children’s Hospital. Written informed consent to participate in this study was provided by the participants’ legal guardian/next of kin.

## Author Contributions

SX and YF performed *in vitro* studies. YF and CY evaluated clinical presentations. TX and XW performed NGS and Sanger sequencing. SJ helped with the manuscript writing. JJ and LX performed *in silico* analyses. WS, HG, and ZH performed data analyses and supervised the study. All authors contributed to the article and approved the submitted version.

## Conflict of Interest

The authors declare that the research was conducted in the absence of any commercial or financial relationships that could be construed as a potential conflict of interest.

## Footnotes

1 https://gnomad.broadinstitute.org/

2 https://varsome.com

## References

[B1] Acker D. W. M. Wong I. Kang M. Paradis S. (2018). Semaphorin 4D promotes inhibitory synapse formation and suppresses seizures in vivo. *Epilepsia* 59 1257–1268. 10.1111/epi.14429 29799628 PMC5990477

[B2] Andermatt I. Wilson N. H. Bergmann T. Mauti O. Gesemann M. Sockanathan S. (2014). Semaphorin 6B acts as a receptor in post-crossing commissural axon guidance. *Development* 141 3709–3720. 10.1242/dev.112185 25209245 PMC6514406

[B3] Capparuccia L. Tamagnone L. (2009). Semaphorin signaling in cancer cells and in cells of the tumor microenvironment–two sides of a coin. *J. Cell Sci.* 122 1723–1736. 10.1242/jcs.030197 19461072

[B4] Cheadle L. Biederer T. (2014). Activity-dependent regulation of dendritic complexity by semaphorin 3A through Farp1. *J. Neurosci.* 34 7999–8009. 10.1523/jneurosci.3950-13.2014 24899721 PMC4044256

[B5] Ellis C. A. Petrovski S. Berkovic S. F. (2020). Epilepsy genetics: clinical impacts and biological insights. *Lancet Neurol.* 19 93–100. 10.1016/s1474-4422(19)30269-8 31494011

[B6] Gant J. C. Thibault O. Blalock E. M. Yang J. Bachstetter A. Kotick J. (2009). Decreased number of interneurons and increased seizures in neuropilin 2 deficient mice: implications for autism and epilepsy. *Epilepsia* 50 629–645. 10.1111/j.1528-1167.2008.01725.x 18657176 PMC2836361

[B7] Genton P. Striano P. Minassian B. A. (2016). The history of progressive myoclonus epilepsies. *Epileptic Disord.* 18 3–10. 10.1684/epd.2016.0834 27621064 PMC5777179

[B8] Hamanaka K. Imagawa E. Koshimizu E. Miyatake S. Tohyama J. Yamagata T. (2020). De novo truncating variants in the last exon of SEMA6B cause progressive myoclonic epilepsy. *Am. J. Hum. Genet.* 106 549–558. 10.1016/j.ajhg.2020.02.011 32169168 PMC7118575

[B9] Kokaia M. (2011). Seizure-induced neurogenesis in the adult brain. *Eur. J. Neurosci.* 33 1133–1138. 10.1111/j.1460-9568.2011.07612.x 21395857

[B10] Li Z. Jagadapillai R. Gozal E. Barnes G. (2019). Deletion of semaphorin 3F in interneurons is associated with decreased GABAergic neurons, autism-like behavior, and increased oxidative stress cascades. *Mol. Neurobiol.* 56 5520–5538. 10.1007/s12035-018-1450-9 30635860 PMC6614133

[B11] Litchfield K. Reading J. L. Lim E. L. Xu H. Liu P. Al-Bakir M. (2020). Escape from nonsense-mediated decay associates with anti-tumor immunogenicity. *Nat. Commun.* 11:3800. 32733040 10.1038/s41467-020-17526-5 PMC7393139

[B12] Neufeld G. Mumblat Y. Smolkin T. Toledano S. Nir-Zvi I. Ziv K. (2016). The role of the semaphorins in cancer. *Cell Adh. Migr.* 10 652–674. 27533782 10.1080/19336918.2016.1197478 PMC5160032

[B13] Perucca P. Bahlo M. Berkovic S. F. (2020). The genetics of epilepsy. *Ann. Rev. Genomics Hum. Genet.* 21 205–230. 32339036 10.1146/annurev-genom-120219-074937

[B14] Poli M. C. Ebstein F. Nicholas S. K. de Guzman M. M. Forbes L. R. Chinn I. K. (2018). Heterozygous truncating variants in POMP escape nonsense-mediated decay and cause a unique immune dysregulatory syndrome. *Am. J. Hum. Genet.* 102 1126–1142. 10.1016/j.ajhg.2018.04.010 29805043 PMC5992134

[B15] Richards S. Aziz N. Bale S. Bick D. Das S. Gastier-Foster J. (2015). Standards and guidelines for the interpretation of sequence variants: a joint consensus recommendation of the american college of medical genetics and genomics and the association for molecular pathology. *Genet. Med.* 17 405–424. 10.1038/gim.2015.30 25741868 PMC4544753

[B16] Tamamaki N. Fujimori K. Nojyo Y. Kaneko T. Takauji R. (2003). Evidence that Sema3A and Sema3F regulate the migration of GABAergic neurons in the developing neocortex. *J. Comp. Neurol.* 455 238–248. 10.1002/cne.10476 12454988

[B17] Tawarayama H. Yoshida Y. Suto F. Mitchell K. J. Fujisawa H. (2010). Roles of semaphorin-6B and plexin-A2 in lamina-restricted projection of hippocampal mossy fibers. *J. Neurosci.* 30 7049–7060. 10.1523/jneurosci.0073-10.2010 20484647 PMC3046408

[B18] Turnbull J. Tiberia E. Striano P. Genton P. Carpenter S. Ackerley C. A. (2016). Lafora disease. *Epileptic Disord.* 18 38–62. 27702709 10.1684/epd.2016.0842 PMC5777303

[B19] Yang J. Yan R. Roy A. Xu D. Poisson J. Zhang Y. (2015). The I-TASSER Suite: protein structure and function prediction. *Nat. Methods* 12 7–8. 10.1038/nmeth.3213 25549265 PMC4428668

[B20] Yazdani U. Terman J. R. (2006). The semaphorins. *Genome Biol.* 7:211. 16584533 10.1186/gb-2006-7-3-211 PMC1557745

