# Supplementary material for: Novel Truncating and Missense Variants in SEMA6B in Patients With Early-Onset Epilepsy
Source: Front Cell Dev Biol. 2021 May 4;9:633819. doi: 10.3389/fcell.2021.633819 (PMC8129541; doi:10.3389/fcell.2021.633819)
Supplement: Supplementary file 1 [file Data_Sheet_1.PDF]

Supplementary Data

Novel truncating and missense variants in *SEMA6B* in patients with early-onset epilepsy

Song Xiaozhen[#1], Yuan Fan[#2,4], Yuan Fang[#3], Lan Xiaoping[1], Jia Jia[5], Xu Wuhen[1], Tang Xiaojun[1], Shen Jun[6] Chen Yucai[2], Zhang Hong[*1], He Guang[*2,4], Wu Shengnan[*1]

Figure S1. Schematic representation of the wild-type *SEMA6B* construct

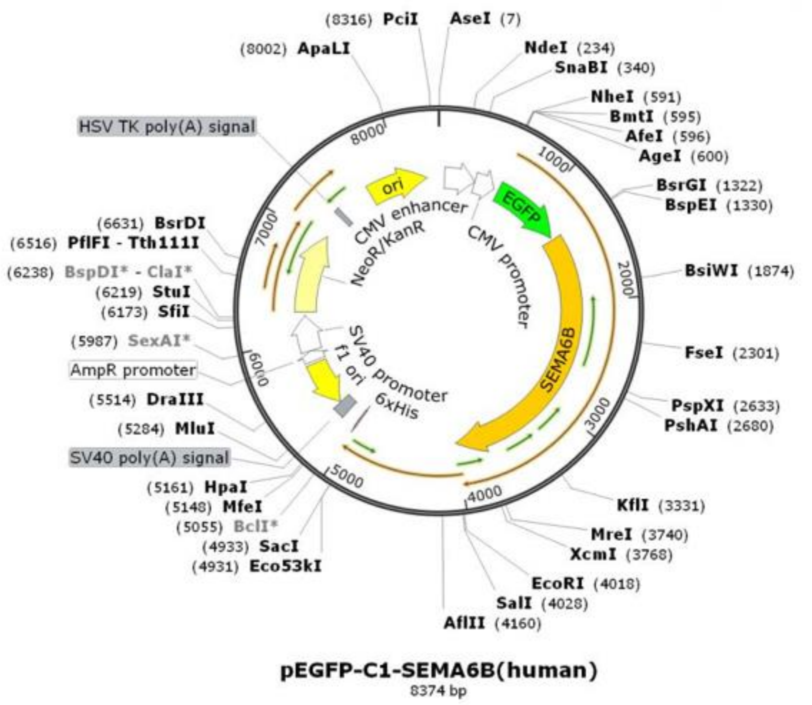

Figure S1. The full-length cDNA of human *SEMA6B* was inserted into the expression vector pEGFP-C1. The plasmid was verified by DNA sequencing. The recombinant plasmid of pEGFP-C1-SEMA6B can be transfected into HEK-293T using Lipofectamine 2000 and expresses N-terminal EGFP tagged SEMA6B fusion protein.

Table S1. Primer sequences of pEGFP-C1-SEMA6B mutants

**Primer**	**Sequence (5' to 3')**	**Purification method**
c.1483G>T_sense	ggcggtggcgagacaTggcagcggctgctgag	ULTRAPAGE
c.1483G>T_antisense	ctcagcagccgctgccAtgtctcgccaccgcc	ULTRAPAGE
c.2056C>T_sense	ctggcgcccctgatgTagaacggctgggccaa	ULTRAPAGE
c.2056C>T_antisense	ttggcccagccgttctAcatcaggggcgccag	ULTRAPAGE
